# [Corrigendum] Human chorionic gonadotropin β regulates epithelial-mesenchymal transition and metastasis in human ovarian cancer

**DOI:** 10.3892/or.2025.8948

**Published:** 2025-07-16

**Authors:** Na Liu, Shu-Min Peng, Guang-Xi Zhan, Jing Yu, Wei-Min Wu, Hao Gao, Xiao-Feng Li, Xiao-Qing Guo

Oncol Rep 38: 1464–1472, 2017; DOI: 10.3892/or.2017.5818

Following the publication of the above article, an interested reader drew to the Editor's attention that, for the scratch-wound assay experiments shown in Fig. 3 on p. 1468, the “ES-2 LV-β-hCG” and “ES-2 siRNA-β-hCG” data panels were apparently the same, suggesting that the same data panel had erroneously been included twice in this figure. Furthermore, β-actin blots featured in Fig. 2C on p. 1467, and certain of the western blot data featured in Fig. 6A on p. 1470, were strikingly similar to western blot data that appeared in papers subsequently published by the same research group in the journals *Cell Cycle* and *Cancer Science*, respectively; finally, the same western blot data had been selected to represent β-catenin and Slug protein bands in Fig. 6A.

After having examined their original data, the authors realized that these figures had been inadvertently assembled incorrectly. The revised versions of Fig. 2 (showing all the correct β-actin data in Fig. 2C), Fig. 3 (showing the correct data for the “ES-2 siRNA-β-hCG” experiment) and Fig. 6 (showing the correct data for the “Slug/ES-2 siRNA-β-hCG” and “Slug/SKOV3 siRNA-β-hCG” protein bands) are shown on the next two pages. Note that the revisions made to these figures do not affect the overall results and conclusions reported in the paper. The authors are grateful to the Editor of *Oncology Reports* for granting them the opportunity to publish this corrigendum, and all the authors agree with its publication; furthermore, they apologize to the readership of the journal for any inconvenience caused.

## Figures and Tables

**Figure 2.**
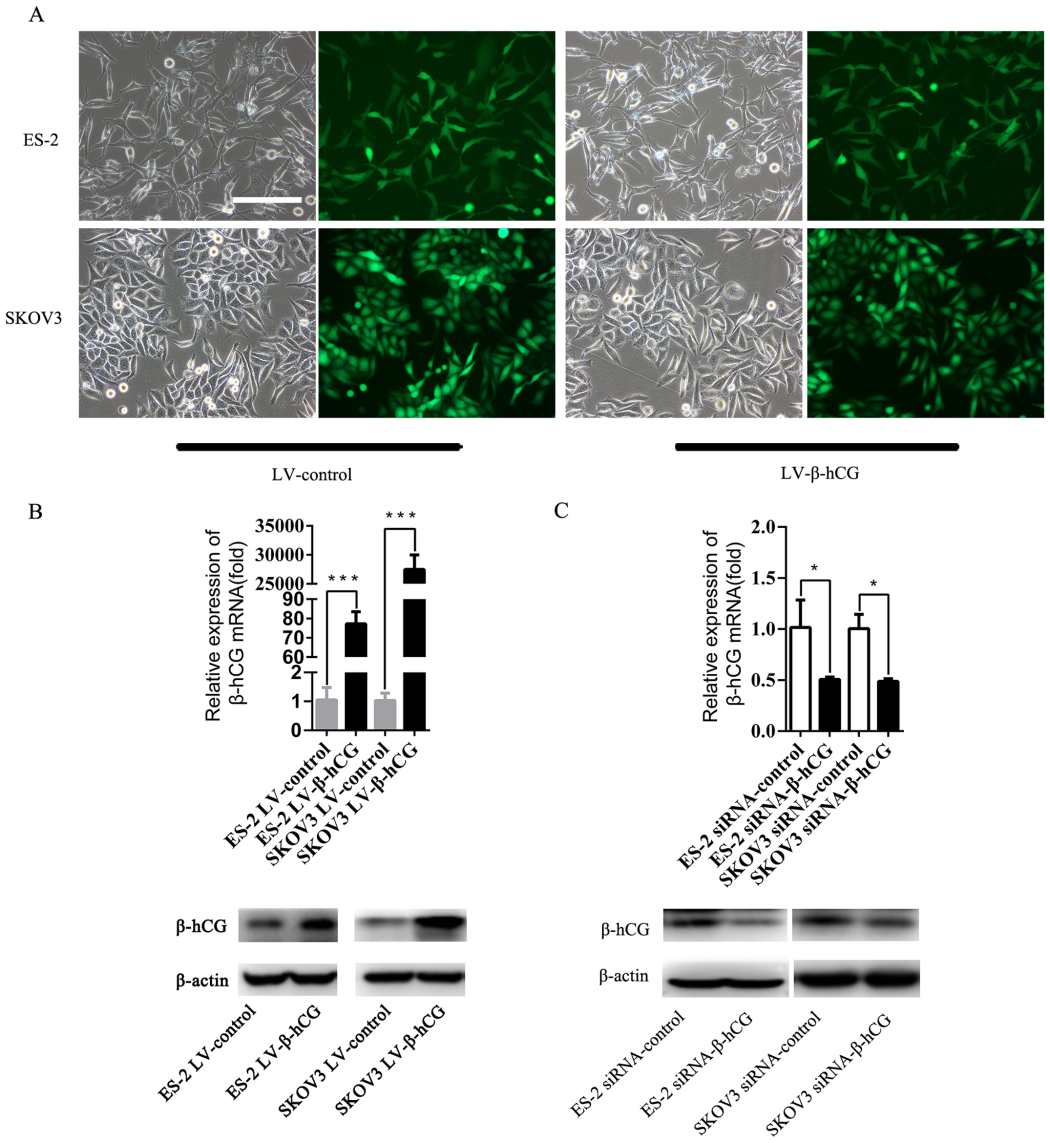
Successful construction of β-hCG-overexpressing and -silenced ovarian cancer cell lines. (A) ES-2 and SKOV3 cells were transfected with LV-vector or LV-β-hCG. Cells were photographed under a fluorescence microscope, using both light and fluorescence. Scale bar, 200 μm. (B) The relative expression level of β-hCG mRNA in β-hCG-overexpressing ES-2 and SKOV3 cells according to RT-PCR analysis and western blotting (***P<0.001). (C) The relative expression level of β-hCG mRNA in β-hCG-silenced ES-2 and SKOV3 cells was determined by RT-PCR analysis and western blotting (*P<0.05).

**Figure 3.**
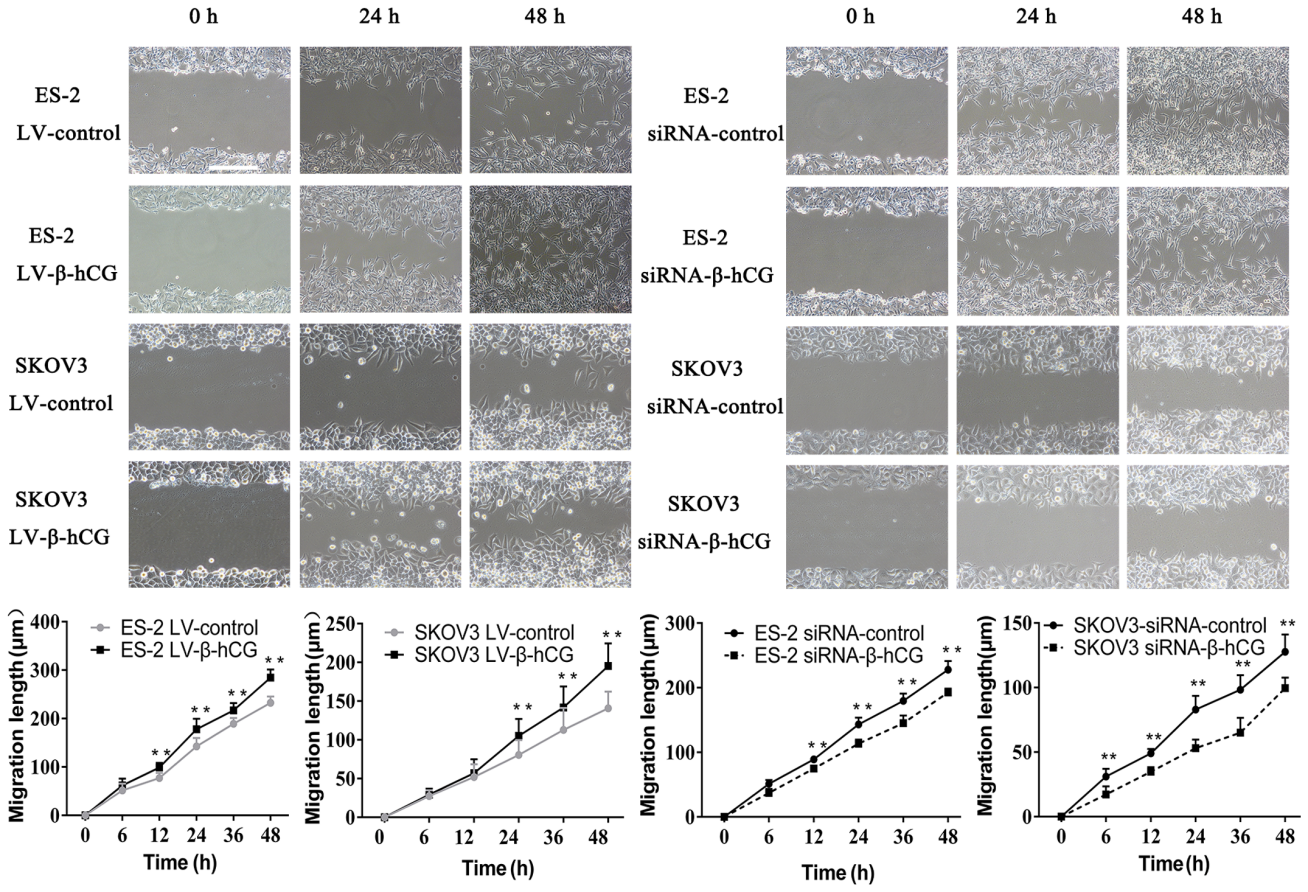
Wound-healing assay showed that β-hCG regulates ovarian cancer cell migration *in vitro* (**P<0.01).

**Figure 6.**
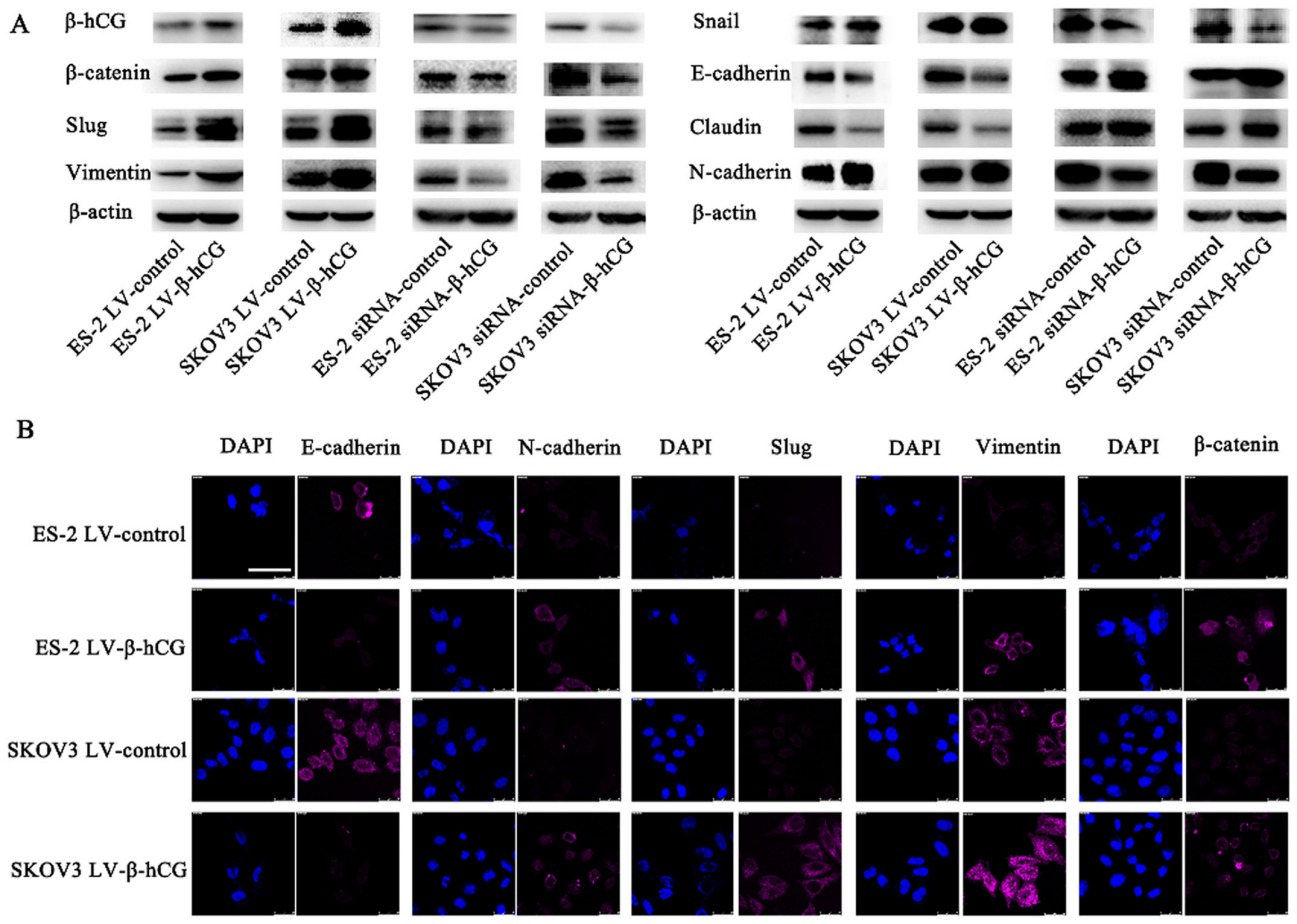
β-hCG induces EMT in ovarian cancer cells. (A) Expression levels of EMT markers in ES-2 and SKOV3 cells transfected with different reagents, as determined by western blotting. (B) Immunofluorescence staining of EMT markers in β-hCG-overexpressing and control cells pictured by laser scanning confocal microscopy. Scale bar, 50 μm.

